# Characterization of *Forestiera tomentosa* Fruit: Proximate Composition, Physicochemical Parameters, Phenolic Content, Antioxidant Capacity, and Toxicological Assessment

**DOI:** 10.3390/molecules31142542

**Published:** 2026-07-22

**Authors:** Salvador Hernández-Estrada, Luis Antonio Ramirez-Contreras, Luis Alfonso Hernández-Villaseñor, Jorge Manuel Silva-Jara, Efigenia Montalvo-González, Zuamí Villagrán, Noé Rodríguez-Barajas, Jorge L. Mejía-Méndez, Carlos Arnulfo Velázquez-Carriles, Martin Zermeño-Ruiz, Luis Miguel Anaya-Esparza

**Affiliations:** 1 Centro Universitario de Los Altos, Universidad de Guadalajara, Tepatitlan de Morelos 47620, Mexico; salvador.hernandez9918@alumnos.udg.mx (S.H.-E.); luis.ramirez3685@alumnos.udg.mx (L.A.R.-C.); luis.hvillasenor@academicos.udg.mx (L.A.H.-V.); blanca.villagran@academicos.udg.mx (Z.V.); noe.rbarajas@academicos.udg.mx (N.R.-B.); 2 Centro Universitario de Ciencias Exactas e Ingenierias, Universidad de Guadalajara, Guadalajara 44430, Mexico; jorge.silva@academicos.udg.mx (J.M.S.-J.); martin.zermeno@academicos.udg.mx (M.Z.-R.); 3 Laboratorio Integral de Investigación de Alimentos, Instituto Tecnológico de Tepic, Tecnológico Nacional de Mexico, Tepic 63175, Mexico; emontalvo@ittepic.edu.mx; 4 Escuela de Ingeniería y Ciencias, Tecnológico de Monterrey, Epigmenio González 500, San Pablo, Santiago de Querétaro 76130, Mexico; mejia.jorge@tec.mx; 5 Centro Universitario de Tlajomulco, Universidad de Guadalajara, Tlajomulco de Zuñiga 45641, Mexico; arnulfo.velazquez@academicos.udg.mx; 6 Centro de Estudios para la Agricultura, la Alimentación y Crisis Climática, Centro Universitario de Los Altos, Universidad de Guadalajara, Tepatitlan de Morelos 47620, Mexico

**Keywords:** wild fruits, underutilized fruits, Oleaceae, bioactive compounds, in silico prediction, sustainable development, potential uses

## Abstract

The demand for sustainable nutrients and bioactive compounds has increased interest in underutilized wild plants. *Forestiera tomentosa*, a Mexican drupe-bearing species, is largely unexplored. This study evaluated the proximate composition, physicochemical and functional properties, phenolic profile, antioxidant capacity, and toxicological safety of *F. tomentosa* fruit. The fruit showed high carbohydrate content [71.94% dry weight (DW)], with notable crude fiber (7.90% DW), protein (7.39% DW), and lipid (4.30% DW) contents. Analysis revealed a mildly acidic pH (5.66), titratable acidity of 0.32%, and total soluble solids of 2.33 °Brix. The fruit powder had a low water activity (0.42) and a favorable water solubility index (56.10%), oil absorption (4.54%), and foaming capacity (19.71%). The fruit contained high levels of soluble phenols (280.42 mg GAE/g DW), flavonoids (98.89 mg CE/g DW), anthocyanins (54.53 mg C3G/g DW), and condensed tannins (94.05 mg CE/g DW). High-performance liquid chromatography identified 20 phenolic compounds, with 3-(4-hydroxyphenyl) propionic acid, syringic acid, catechin, epicatechin, and gallocatechin being predominant. The fruit showed significant radical scavenging and reducing potential (DPPH, ABTS, and FRAP). Toxicological evaluation using the *Artemia salina* bioassay showed a 100% survival rate across all concentrations, indicating no acute toxicity. In silico ADMET predictions revealed favorable pharmacokinetic properties, including high intestinal absorption and compliance with Lipinski’s rule of five. These findings position *F. tomentosa* as a promising, non-toxic source of functional ingredients for the food, nutraceutical, and pharmaceutical industries, supporting biodiversity conservation and sustainable resource utilization. Further studies are needed to evaluate the potential health benefits of this fruit in vitro and in vivo.

## 1. Introduction

Native and edible fruits have attracted increasing scientific interest because they represent valuable sources of nutrients and bioactive phytochemicals with potential applications in the food, nutraceutical, cosmetic, and pharmaceutical industries. Despite this potential, many of these species remain undervalued due to limited scientific information on their chemical composition, phytochemical profiles, and biological properties, which restricts their integration into value chains and conservation strategies [1]. Therefore, the comprehensive characterization of underutilized wild fruits is essential to support their sustainable use, promote biodiversity conservation, and identify new plant resources with functional potential [2].

The family Olaceae comprises a moderately sized assemblage of woody plants, encompassing 28 genera, several of which are notably used to produce oils and fragrances. Nonetheless, this family also includes a lesser-known genus, *Forestiera* [3]. The genus *Forestiera* comprises small deciduous and semi-deciduous trees and shrubs distributed across the United States, Mexico, Central America, and South America, thriving in vegetation types characterized by distinct seasonal patterns. Furthermore, 14 species of this genus have been identified, with the majority (51%) located in Mexico [4,5,6]. The most frequently reported *Forestiera* species include *F. neomexicana* [7], *F. isabelae* [8], *F. acuminata* [9], *F. veracruzana* [6], *F. angustifolia*, *F. pubescens* [3], and *F. tomentosa* [10]. It is important to highlight that those studies on *Forestiera* species have focused on its morphology, palynology, and wood anatomy [5], whereas information regarding the chemical composition of its fruits remains scarce.

*Forestiera tomentosa* is a species native to and currently known only from Mexico, where it is distributed across several central and western states, including Aguascalientes, Colima, Guanajuato, Guerrero, Hidalgo, Michoacan, Oaxaca, Puebla, Queretaro, San Luis Potosi, Zacatecas, and Jalisco [11]. It is known as acebuche, arcibuche, aceitunilla, or granjeno and is a shrub or small tree (up to 6 m tall) with smooth, gray-to-blackish bark with leaves that are narrow to broadly elliptic, sometimes varying to oblong, lanceolate, or oblanceolate; moreover, it produces fruit of an ellipsoid shape of 7 to 11 mm long and 4 to 8 mm wide, and epicarp of a purple-blackish or deep-blue color when ripe, containing one seed of 4 mm, while its endocarp becomes rigid with maturation [12]. Additionally, the small drupes serve as a food source for local fauna, and these wild fruits are edible [13] and consumed by local people fresh and in salads, with an astringent flavor like that of red wine made from grapes (personal communication). To the best of our knowledge, no studies have comprehensively characterized the nutritional composition, physicochemical properties, phenolic profile, antioxidant capacity, or toxicological characteristics of *F. tomentosa* fruit. This knowledge gap highlights the need for future studies to develop sustainable processing strategies that maximize the utilization of this unexplored native fruit and support its potential applications in the food, nutraceutical, cosmetic, and pharmaceutical industries.

In this context, aligning with the shift from traditional medicine–food homology to a modern evidence-based paradigm [14], the aim of this study was to provide the first scientific characterization of *F. tomentosa* fruit by evaluating its proximate composition, physicochemical properties, phenolic profile, antioxidant capacity, and toxicological profile.

## 2. Results

**Figure 1:**
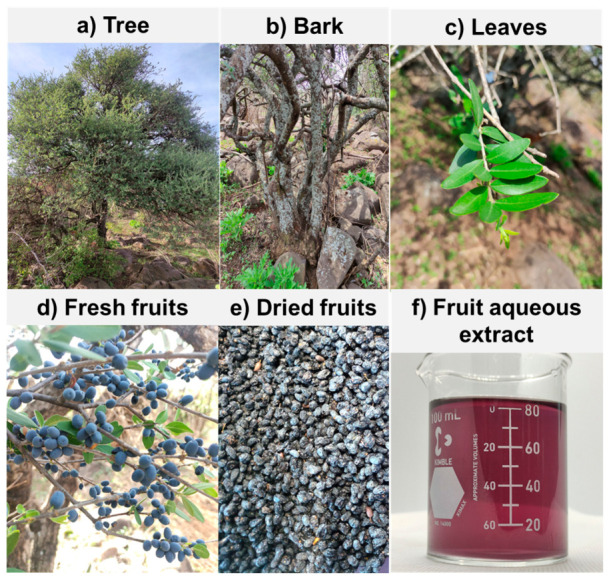
Visual appearance of *Forestiera tomentosa* tree (**a**), bark (**b**), leaves (**c**), fresh fruits (**d**), dried fruits (**e**), and fruit aqueous extract (**f**).

Figure 1 illustrates the visual appearance of the *Forestiera tomentosa* tree, including bark, leaves, and fresh fruits (Figure 1a–d). Moreover, dried fruits (Figure 1e) and fruit aqueous extract (Figure 1f) are also shown.

### 2.1. Proximate Composition

The proximate composition of *F. tomentosa* fruit is shown in Table 1. The fresh fruit contains 67.85% moisture, whereas the dried fruit contains only 12.04%. The dried fruits are characterized by their concentration of available carbohydrates (71.94%, calculated by difference), including 21.40% reducing sugars and 7.90% crude fiber, followed by protein (7.39%), lipids (4.30%), and ash (4.33%).

### 2.2. Physicochemical Parameters and Functional Properties

Table 2 presents the physicochemical and functional properties of *F. tomentosa* fruits. The physicochemical parameters of the fresh fruits included a TSS content of 2.33 °Brix, a pH of 5.66, and a titratable acidity (TA) of 0.32%, yielding a TSS/TA ratio of 7.28. The color parameters of fresh fruits of *F. tomentosa* indicated a luminosity of 26.26, with positive *a* (1.01) and negative *b* (−15.30) coordinates. Additionally, the dried fruit powder exhibited a water activity of 0.40. The functional properties of the dried fruit included a water solubility index of 56%, a water absorption index of 1.80 g g^−1^, and a swelling power of 5.53%. Additionally, the fruit powder exhibited an oil absorption index of 2.48 g g^−1^, a foaming capacity of 19.71%, and an emulsifying capacity of 2.61%.

### 2.3. Phenolic Compounds and Antioxidant Capacity

The fruits of *F. tomentosa* contained 280 mg GAE/g of soluble phenols, 98.89 mg CE/g of flavonoids, 54.53 mg C3G/g of anthocyanins, and 94.95 mg CE/g of condensed tannins. Furthermore, *F. tomentosa* fruits exhibited antioxidant activity by DPPH (14.59 mmol TE/g), ABTS (12.04 mmol TE/g), and FRAP (1.92 mmol TE/g) assays (Table 3).

Additionally, twenty phenolic compounds were identified from *F. tomentosa* fruit by HPLC-DAD, including phenolic acids and flavonoids such as gallic, protocatechuic, neochlorogenic, 3,4-Dihydroxyphenylacetic, 4-Hydroxybenzoic, chlorogenic, 4-Hydroxyphenylacetic, vanillic, syringic, 3-(4-hydroxyphenyl) propionic, p-coumaric, trans-ferulic, ellagic, and salicylic acids, gallocatechin, catechin, epicatechin, rutin, and naringenin (Table 4). The most abundant compounds were 3-(4-hydroxyphenyl) propionic acid (172,711 µg/100 g), syringic acid (55,791 µg/100 g), catechin (29,630 µg/100 g), epicatechin (25,387 µg/100 g), gallocatechin (23,693 µg/100 g), and 4-Hydroxyphenylacetic acid (9229 µg/100 g).

### 2.4. Toxicity Bioassay in Artemia salina

Table 5 shows the results of the *Artemia salina* toxicity bioassay, in which the survival (%) of larvae was measured after 24 and 48 h of exposure to *F. tomentosa* extract at different concentrations. In all tested concentrations (1000–62.5 µg/mL), the survival rate of *A. salina* was 100%, like the synthetic seawater (negative control). On the other hand, the *A. salina* larvae exposed to sodium hypochlorite (positive control) died during the first 24 h of exposure.

### 2.5. In Silico Evaluation of Drug-likeness and Toxicity of Main Phenolic Compounds Identified from Forestiera tomentosa Fruit

Table 6 lists the physicochemical parameters (molecular weight, the number of hydrogen-bond acceptors and hydrogen-bond donors, lipophilicity, and the number of violations of Lipinski’s rule of five) of the main phenolics (syringic acid, catechin, epicatechin, gallocatechin, 3-(4-Hydroxyphenyl) propionic acid, and hydroxyphenylacetic acid) found in *F. tomentosa* fruit by HPLC-DAD.

**Figure 2:**
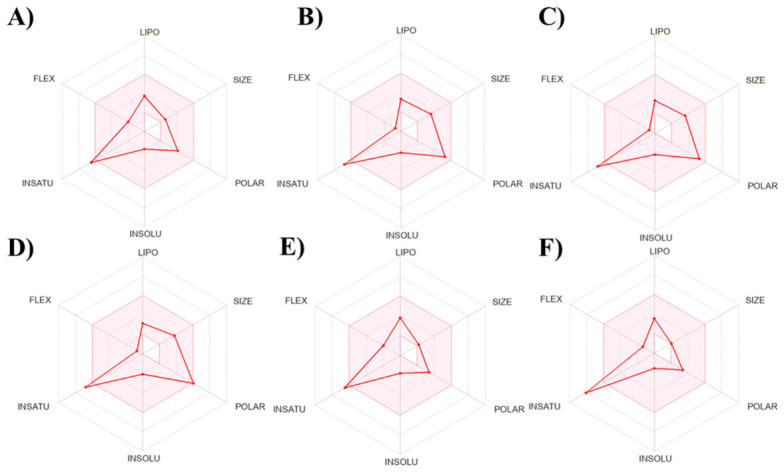
The radar image obtained from SwissADME for the top 6 compounds present in the extract: (**A**) syringic acid, (**B**) catechin, (**C**) epicatechin, (**D**) gallocatechin, (**E**) 3-(4-Hydroxyphenyl) propionic acid, and (**F**) 4-hydroxyphenylacetic acid.

These compounds exhibited lipophilicity values ranging from 0.88 to 1.54 Log *P*_o/w_, and none violated any of Lipinski’s rules, except for gallocatechin, which violated one (NH or OH > 5). Furthermore, these compounds exhibited good physicochemical properties, including lipophilicity, size, polarity, insolubility, unsaturation, and flexibility (Figure 2). Lipinski’s rules are based on physicochemical parameters designed to assess chemical compounds and determine their pharmacokinetic properties. Smaller molecules, ideally with a molecular weight of 500 g/mol or less, and those with no more than 10 hydrogen-bond acceptors, up to 5 hydrogen-bond donors, and a lipophilicity of 5 or less generally cross cell membranes more efficiently. Consequently, they are considered superior drug candidates compared with larger molecules that exhibit excessive hydrogen bonding. In this evaluation, most of the compounds studied exhibited optimal physicochemical and pharmacokinetic properties, except for gallocatechin, which had more than five hydrogen-bond donors.

**Figure 3:**
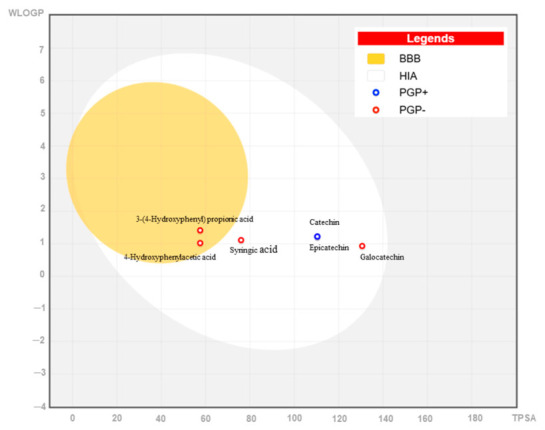
Egg plot from SwissADME showed the water partition coefficient (WlogP) versus the topological polar surface area (TPSA) for the six compounds with the highest presence in *F. tomentosa*.

On the other hand, ADME characteristics are associated with the potential to achieve favorable oral bioavailability (OB), human intestinal absorption (HIA), and effective traversal of the blood–brain barrier (BBB) (Table 6). Notably, 3-(4-hydroxyphenyl)propionic acid (0.79), syringic acid (0.73), and 4-hydroxyphenylacetic acid (0.79) were distinguished by their OB values, whereas gallocatechin (0.35), epicatechin (0.39), and catechin (0.40) exhibited low OB values. For their part, syringic acid (0.99), catechin (0.99), and epicatechin (0.99) demonstrated the highest HIA values, compared with gallocatechin (0.98), 3-(4-hydroxyphenyl)propionic acid (0.98), and 4-hydroxyphenylacetic acid (0.95). Furthermore, 3-(4-hydroxyphenyl)propionic acid (0.45), 4-hydroxyphenylacetic acid (0.45), and syringic acid (0.43) are more likely to cross the BBB than gallocatechin (0.09), epicatechin (0.13), and catechin (0.14) (Figure 3).

Additionally, we assessed the toxicological properties of the six most prevalent compounds identified in *F. tomentosa* (Table 7). The clinical toxicity values ranged from 0.01 to 0.08, whereas the potential to inhibit the human ether-à-go-go-related gene (hERG) ranged from 3.71 × 10^−3^ to 0.30. Notably, syringic acid (0.69) exhibited a higher propensity to induce hepatotoxicity. All compounds showed a low probability of causing mutagenicity (0.05 to 0.34); however, catechin (0.34), epicatechin (0.33), and gallocatechin (0.33) exhibited the highest values. Similar trends were observed for carcinogenicity, with all compounds exhibiting a low probability of inducing carcinogenicity, ranging from 0.05 to 0.22; 4-hydroxy-phenylacetic acid (0.19) and 3-(4-hydroxy-phenyl) propionic acid (0.22) had higher values. Regarding the prediction of LD_50_ values (in humans), 4-hydroxyphenylacetic acid (1550 mg/kg), syringic acid (1700 mg/kg), and 3-(4-hydroxy-phenyl) propionic acid (2000 mg/kg) showed the lowest values, whereas catechin, epicatechin, and gallocatechin showed the highest values (10,000 mg/kg); moreover, these compounds showed a toxicity class ranging from four to six.

Finally, we performed molecular docking simulations of the six compounds with the three isoforms of the cytochrome P450 enzyme (CYP3A4, CYP2D6, and CYP2E1). Table 8 shows the binding scores (ΔGs) for the three target proteins. A ΔG (kcal/mol) < 0 suggests the possibility of spontaneous binding of the six compounds to the proteins. The results showed that the ΔG values for the three proteins were negative, indicating that all compounds spontaneously bound to the proteins.

**Figure 4:**
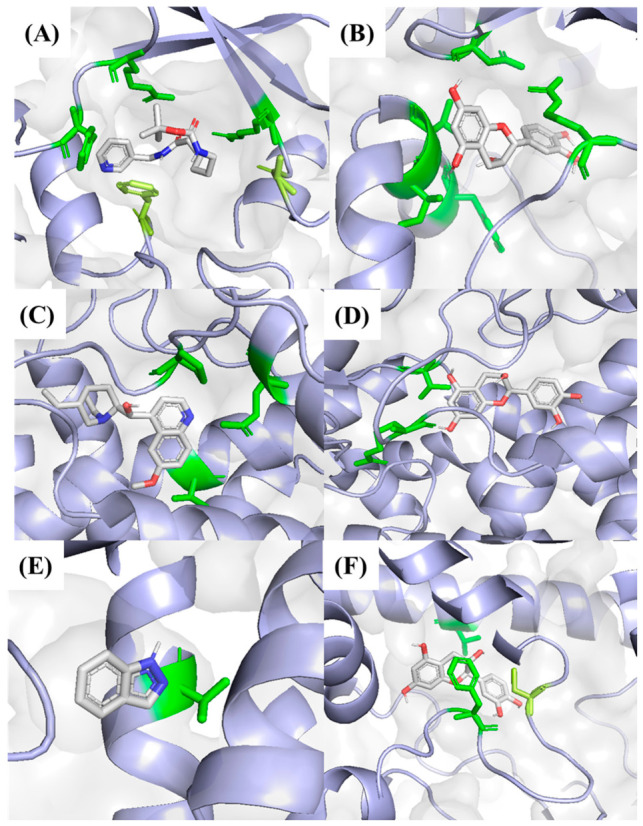
3D representation of the complexes: (**A**) CYP3A4-Tert-Butyl {6-Oxo-6-[(Pyridin-3-Ylmethyl)amino]hexyl}carbamate; (**B**) CYP3A4–epicatechin; (**C**) CYP2D6–quinine; (**D**) CYP2D6-epicatechin; (**E**) CYP2E1–indazole; (**F**) CYP2E1–catechin. The prominent green residues represent conventional hydrogen bonds, while the lime-green residues represent a carbon–hydrogen bond in (**A**) and a pi-donor hydrogen bond in (**F**).

**Figure 5:**
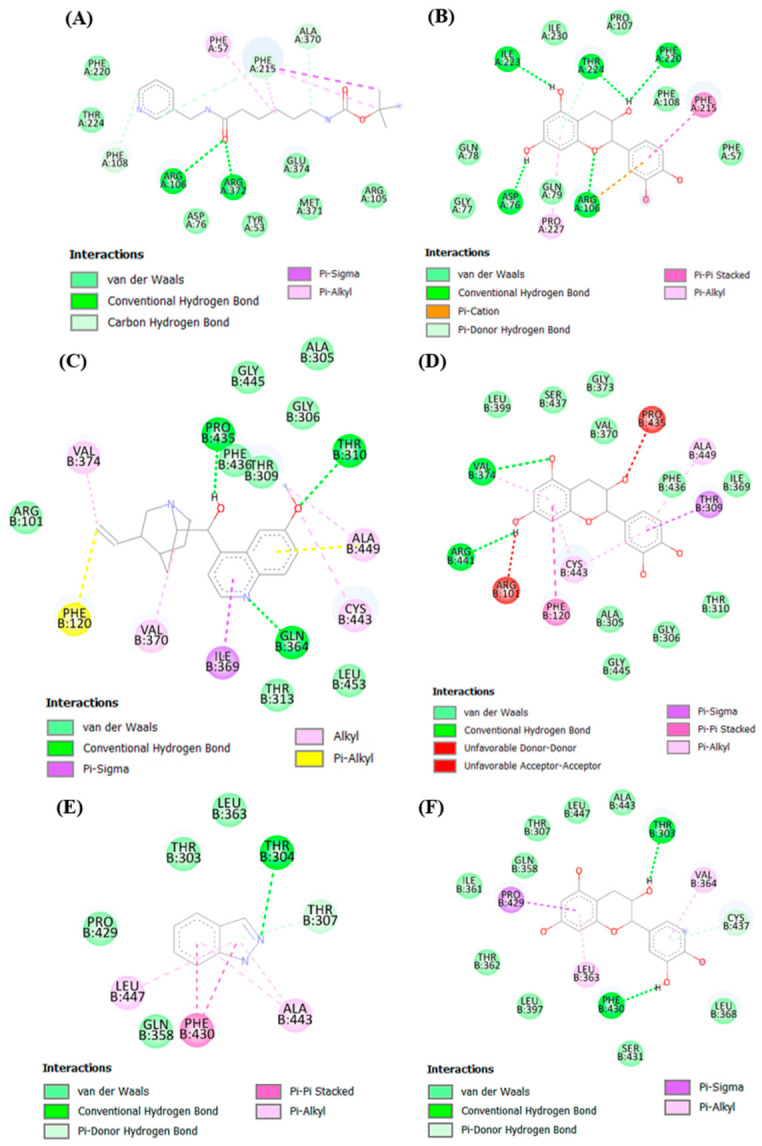
Favorable interactions of complexes: (**A**) CYP3A4-Tert-Butyl {6-Oxo-6-[(Pyridin-3-Ylmethyl)amino]hexyl}carbamate; (**B**) CYP3A4–epicatechin; (**C**) CYP2D6–quinine; (**D**) CYP2D6–epicatechin; (**E**) CYP2E1–indazole; (**F**) CYP2E1–catechin.

Figure 4 illustrates the 3D molecular docking between the target proteins and the compounds with the most favorable values, including controls for each enzyme. Furthermore, compared to the CYP3A4 inhibitor, tert-butyl {6-Oxo-6-[(Pyridin-3-ylmethyl)amino]hexyl}carbamate (−7.5 kcal/mol), epicatechin (−8.8 kcal/mol), gallocatechin (−8.4 kcal/mol), and catechin (−8.3 kcal/mol) showed better affinity. However, no compound showed greater affinity than the CYP2D6 control, quinine (−9.0 kcal/mol). All compounds showed better affinity than the CYP2E1 inhibitor indazole (−6.0 kcal/mol). In the CYP3A4–epicatechin complex, four hydrogen bonds (ASP^76^, ARG^106^, PHE^220^, ILE^223^, and THR^224^) were found, while in the control (CYP3A4-Tert-Butyl {6-Oxo-6-[(Pyridin-3-Ylmethyl) amino] hexyl} carbamate)) five hydrogen bonds were detected (ARG^106^, PHE^108^, PHE^215^, ALA^370^, ARG^372^). In the case of the CYP2D6–epicatechin complex, two hydrogen bonds were detected (VAL^374^ and ARG^441^), whereas in the control (CYP2D6–quinine), three hydrogen bonds were detected (THR^310^, GLN^374^, and PRO^415^). On the other hand, in the CYP2E1–catechin complex, there are two hydrogen bonds (THR^303^ and PHE^430^), while in its respective control (CYP2E1–indazole), only one hydrogen bond was detected (THR^304^) (Figure 5).

## 3. Discussion

The valorization of wild fruits has emerged as a critical strategy for identifying novel sources of nutrients and bioactive compounds while promoting the sustainable utilization of underexplored natural resources [2]. *Forestiera tomentosa*, an endemic Mexican species of the Oleaceae family, has remained scientifically overlooked, with its utilization traditionally limited to wood [5]. The fruits are small, fleshy drupes with an ellipsoid–oblong morphology, measuring approximately 6–10 mm in length and 3.5–5 mm in diameter. At physiological maturity, they exhibit a characteristic purple-to-deep-blue coloration, often covered by a pruinose layer that imparts a matte appearance [13]. According to local inhabitants, these fruits are edible and possess a distinctive astringent, wine-like flavor. However, no information about these fruits is available in the scientific literature.

Therefore, the present study provides novel insights into the proximal, physicochemical, phenolic, antioxidant, and toxicological profiles of *F. tomentosa* fruit, a genus that remains largely underexplored compared to other members of the Oleaceae family. In this context, comparable data for other *Forestiera* species are limited or nonexistent, further emphasizing the novelty and relevance of this study. In this context, in the absence of prior research on *Forestiera tomentosa* fruits or those of other *Forestiera* species, the findings were primarily compared with those reported for olive (*Olea europaea*) fruits, which are the most extensively studied both economically and scientifically within the Oleaceae family [16], to which *F. tomentosa* belongs [5]. Nonetheless, other wild edible drupes were selected for additional comparisons.

The proximate composition of *F. tomentosa* fruit suggested that this wild drupe may be a good source of carbohydrates, with appreciable amounts of crude fiber, protein, and fat. The composition of this fruit is comparable to that of other wild drupes, such as *Prunus spinosa*, which has been reported to contain high carbohydrate levels (88.51% DW) and moderate protein (6.46% DW) and fat (4.11% DW) contents [17,18]. Compared to olives, *F. tomentosa* showed comparable values for protein (1.45–5.99% DW) and crude fiber (1.29–3.91% DW) [19,20]; however, not for fat content, which typically ranges from 17 to 30% lipids depending on the cultivar [20]. Furthermore, the moisture content in fresh fruits of *F. tomentosa* is like that of olive fruits, with values ranging from 58 to 63% [18,20]. These results suggest that *F. tomentosa* fruit is a nutritive wild drupe with potential for both consumption and food production.

The physicochemical profile of *F. tomentosa* fruits, characterized by a mildly acidic pH, moderate titratable acidity, and low total soluble solids, results in a low TSS/TA ratio, a key factor in fruit flavor perception. Low TSS/TA ratios are consistently associated with a sour, non-sweet profile [21]. A comparable pH value (5.03–5.66) was reported in five Turkish olive fruit varieties [22]. Fresh olives are characterized by low sugar content and relatively high levels of organic acids and phenolic compounds, which contribute to their bitter and astringent taste rather than sweetness [23]. Conversely, *Vaccinium corymbosum* fruits typically exhibited a TSS/TA ratio higher than 10, indicating a sweeter flavor profile [24]. Furthermore, the purple-blackish color of *F. tomentosa* fruit is indicative of anthocyanin accumulation, like that of *Vaccinum corymbosum* fruits [25].

From a technological perspective, the dried fruit exhibited a low water activity, suggesting high microbiological stability and a reduced rate of degradative chemical reactions, thereby facilitating long-term storage without extensive use of preservatives [26]. For its part, the functional properties of *F. tomentosa* dried fruit, particularly its high water-solubility index, oil absorption, and foaming capacity, suggest its applicability as a functional ingredient in diverse food systems. High oil absorption capacity is commonly associated with enhanced flavor retention and mouthfeel in formulated foods, due to the binding of lipophilic compounds [27]. Likewise, solubility plays a critical role in the development of beverage formulations, where rapid dispersion and homogenization integration are essential for product stability and acceptance [28]. In addition, foaming capacity contributes to the formation and stabilization of air bubbles in bakery and aerated food systems, thereby directly influencing texture and structural properties [29]. The physicochemical and functional properties of fresh and dried *F. tomentosa* fruit can be beneficial for the development of foods as functional ingredients.

One of the most remarkable findings of this study is the high concentration of soluble phenols and flavonoids, including anthocyanins. These values are significantly higher than those reported for five green olive varieties (total phenolic content ranging from 8.74 to 18.8 mg GAE/g, total flavonoids values of 6.25 to 13.42 mg CE/g, and anthocyanins ranging from 0.001 to 0.541 mg C3G/g) [20] and other widely consumed superfruits such as *Vaccinum corymbosum* fruits with a polyphenol content of 5.9 mg GAE/100 g (DW) and anthocyanins with 1.7 mg GAE/100 g (DW) [30]. These elevated phenolic concentrations strongly correlate with the high antioxidant capacity observed across DPPH, ABTS, and FRAP [31]. These results suggested that *F. tomentosa* fruit may be an important source of antioxidant compounds.

The HPLC-DAD analysis revealed a diverse phenolic profile, including phenolic acids and flavonoids, with 3-(4-hydroxyphenyl) propionic acid and syringic acid as the predominant compounds. Additionally, the high levels of flavan-3-ols (catechin, epicatechin, and gallocatechin) could explain the astringent sensory perception of the fruit, as in other plant-based foods rich in catechin and tannins, including tea, cocoa, and wine [31,32]. These compounds have been associated with antioxidant, anti-inflammatory, and cardioprotective effects, playing a key role in modulating oxidative stress and supporting human health [33].

The absence of acute toxicity in the *Artemia salina* bioassay (at the evaluated concentrations) indicates low acute toxicity and provides strong preliminary evidence for the safety of *F. tomentosa* fruit [34]. This bioassay is widely recognized as a reliable screening tool for cytotoxicity and correlates well with more complex biological systems [35,36]. The application of computational biology and bioinformatics tools not only conserves time and resources but also accelerates and enhances the generation of results from in vitro and in vivo experiments, while facilitating future advancements [37]. The in silico analysis of phytochemical compounds through ADMET (absorption, distribution, metabolism, excretion, and toxicity) profiling has emerged as a valuable method for assessing drug-like characteristics and pharmacokinetic properties [38]. The in silico ADMET analysis further supports the safety and potential bioavailability of the identified major phenolic compounds. High predicted human intestinal absorption (HIA > 0.95) indicates good bioavailability, a critical factor for their biological efficacy. Moreover, compliance with Lipinski’s rule of five indicates favorable drug-likeness properties [39]. Although syringic acid exhibited a higher theoretical propensity for hepatotoxicity, the predicted LD_50_ values and toxicity classes indicate that all evaluated compounds are generally safe for consumption [40,41].

These results bridge the gap between traditional knowledge and scientific validation, positioning *F. tomentosa* as a promising, non-toxic source for the food and nutraceutical industries. Although *F. tomentosa* fruits are edible to local inhabitants, proper identification of the plant and moderation in consumption are required, especially if people are not accustomed to eating wild drupes.

Conversely, components in medicinal plant extracts can affect drug pharmacokinetics, potentially leading to toxicity or a lack of therapeutic effect, depending on whether the drug reaches excessively high or insufficient levels in tissues [42]. Molecular docking could help to explore the interactions between plant-derived chemical compounds and target molecules involved in critical metabolic processes, such as xenobiotic pathways [43]. Cytochrome P450 enzymes are part of a large family of proteins that are crucial for the metabolism of both foreign substances and naturally occurring compounds in the body [44]. In this study, molecular docking analysis was used to elucidate the interactions between compounds from *F. tomentosa* and cytochrome P450 isoforms. While all compound–protein interactions demonstrated low ∆G values, epicatechin and catechin were particularly notable for their binding affinities to the CYP3A4, CYP2D6, and CYP2EI isoforms. CYP3A4 is the most widely expressed CYP isoform and plays a pivotal role in pharmacology [45]. In an in vitro study, the amino acid THR^224^ has been identified as significant for substrate binding in the mechanism of action of CYP3A4 [46]. In this study, epicatechin interacts with THR^224^ through a hydrogen bond, which may contribute to its stability in binding to the enzyme. Although accounting for only 2–4% of total hepatic CYP content, CYP2D6 is essential for drug metabolism and processes approximately 20% of widely used medications [47]. In contrast to the results obtained in this study using in silico tools, in vitro research conducted by Satoh et al. [48] observed that catechins and epicatechins from green tea are weak inhibitors of CYP3A4 and CYP2D6. Meanwhile, gallate catechins were found to have a greater inhibitory effect. Additionally, site-directed mutagenesis assays have identified PHE^120^ as a fundamental amino acid in substrate orientation within the active site and therefore in the regiospecificity of CYP2D6 [49]. This study shows binding at this amino acid through π-alkyl and π-π stacked interactions by quinidine and epicatechin, respectively. CYP2EI is notable for its contribution to liver homeostasis and its ability to activate procarcinogens and convert certain drugs, including paracetamol and anesthetics [50]. The docking results revealed interactions between both imidazole (control) and catechin with THR^303^. The relevance of THR^303^ in the substrate recognition process, which is part of the active site of CYP2E1, has been demonstrated in both in silico and in vitro studies [51,52]. Although there is a lack of specific information on the interaction between catechin and CYP2E1, catechin concentrations in the range of 50–250 μM have been observed to exhibit both antioxidant and pro-oxidant effects in microsomal membranes expressing active CYP2E1 [53]. In contrast, epicatechin did not inhibit CYP2E1 and CYP3A4 in the membrane fraction of genetically modified *Salmonella* Typhimurium [54]. In addition, similar in silico approaches have been successfully employed to assess the potential of other fruits, such as goji berries, against aging-related diseases, highlighting the applicability of this method for prioritizing bioactive compounds in understudied species [55].

These findings open new directions for exploring the role of each compound present in *F. tomentosa* and the synergies among them, or with medications, with the aim of developing or using safe products from this plant material.

## 4. Future Prospects

Recent perspectives emphasize that the successful translation of underutilized food resources into value-added products requires an evidence-based approach integrating comprehensive chemical characterization, biological validation, safety assessment, sustainable processing, and value-chain development [56]. In this context, the findings of the present study provide an initial scientific foundation for exploring the future applications of *F. tomentosa* fruit, which may include the following strategic areas:

1. Food industry applications: The fruit can be used as a functional ingredient in developing food products, due to its bioactive compound content and antioxidant capacity. Aqueous fruit extracts can be used as a natural colorant in solid and liquid foods. Furthermore, this fruit can be explored to make fermented and non-fermented beverages.
2. Cosmetic and personal-care products: The high concentrations of flavonoids and tannins position *F. tomentosa* fruit as a candidate for developing cosmetic products, focusing on anti-aging and UV-protection products. Moreover, this fruit could be used as a sustainable alternative to synthetic dyes in makeup products.
3. Pharmaceutical and phytochemistry discovery: The identification of 20 phenolic compounds, particularly the abundance of 3-(4-Hydroxyphenyl) propionic acid and catechins, opens avenues for drug discovery. For example, plant-derived supplements or nutraceuticals with antidiabetic, anti-inflammatory, or antihypertensive properties, supported by further in silico, in vitro, in vivo, and clinical studies.
4. Sustainable pigment source: The deep blue-blackish color of the fruit and its anthocyanin content are clear indicators of its potential as a natural pigment source.

Future efforts should optimize anthocyanin extraction for use as a pH-sensitive colorant in biosensor development to detect microbial changes in food products.

5. Biopesticides, biofertilizers, and plant growth promotion: Bioactive compounds from *F. tomentosa* fruit could serve as natural biopesticides and biofertilizers, supporting sustainable crop protection and soil health. Additionally, allelopathic effects may be harnessed for weed control and improved plant growth, encouraging agroecological practices and reducing reliance on synthetic chemicals.
6. Green synthesis of nanoparticles: The reducing power and stabilizing capacity of the phenolic compounds found in *F. tomentosa* suggest its potential as a sustainable biorefinery for the green synthesis of metallic nanoparticles (i.e., Ag, Au, and Zn). These green-synthesized nanoparticles could exhibit antimicrobial and catalytic properties, offering an eco-friendly alternative to conventional chemical reduction methods.
7. Comprehensive bioactive profiling: Using advanced omics technologies such as metabolomics, genomics, and transcriptomics allows for a more thorough understanding of the complete range of bioactive compounds in *F. tomentosa* fruit. These methods help identify new metabolites, clarify biosynthetic pathways, and aid in future breeding or biotechnological efforts to improve phytochemical yields.
8. Preservation of traditional knowledge: Since there are no documented traditional uses of *F. tomentosa*, conducting ethnobotanical surveys is crucial. Recording any local or indigenous knowledge about the fruit will enhance scientific understanding, help preserve cultural heritage, and guide sustainable bioprospecting efforts.
9. Sustainable value-chain development: The future commercialization of *F. tomentosa* fruit and its derivatives could support rural livelihoods, provided that sustainable harvesting protocols, cultivation systems, and conservation strategies are established to prevent overexploitation of natural populations. Such an approach would facilitate the development of resilient value chains while contributing to biodiversity conservation and the circular bioeconomy.

Although the findings of this study highlight the promising potential of *F. tomentosa* fruit for multiple industrial applications, the species is currently known only from wild populations in Mexico. Therefore, any future exploitation of this resource should be preceded by ecological assessments, population monitoring, and the development of sustainable harvesting protocols. Furthermore, studies on domestication, agronomic management, and cultivation practices are needed to ensure a reliable and sustainable supply of raw materials while preserving natural populations and maintaining biodiversity. Collectively, these actions will be essential for supporting the responsible valorization of *F. tomentosa* fruit, ensuring that its future commercial development contributes to biodiversity conservation, sustainable resource management, rural livelihoods, and the advancement of the circular bioeconomy.

## 5. Materials and Methods

### 5.1. Forestiera tomentosa Fruits

*Forestiera tomentosa* fruits (4.5 kg) were collected from wild trees located in Valle de Guadalupe, Jalisco, Mexico (coordinates: 102°36′45″ W, 20◦57′15″ N; altitude, 1973 m.a.s.l). The fruits were manually harvested in early June 2025 and transported from the University of Guadalajara to the Research Institute of Medical Sciences of the Center of Los Altos, on the same day of collection. Then, the fruits were manually cleaned of impurities, washed, and disinfected with a sodium hypochlorite solution at 200 ppm for 10 min. Fresh fruit samples were analyzed immediately after sanitization for all physicochemical determinations that require fresh fruit to minimize postharvest changes. The remaining fruits were dried in a forced-convection oven (LUZEREN^®^, DGH9070A, ISSE LABS S.A de C.V., Mexico City, Mexico) at 50 °C for 72 h. Afterward, fruits were ground using a Nutribullet^®^ food processor (NB-101, Pacoima, CA, USA) and preserved in resealable metallic bags at ambient temperature until use. The plant material was identified by the biologist Juan Jose Montiel-Avila at Vivero Maculxóchitl (La Purificación, Texcoco, Estado de México, Mexico). The samples were deposited with the voucher number 1812LEZ.

### 5.2. Proximate, Physicochemical, and Functional Characterization

Proximate analysis was performed in accordance with AOAC guidelines [57]. Crude protein content was determined using the Kjeldahl method, employing a conversion factor of 6.25 (AOAC method 968.06). Lipid content was assessed via the Soxhlet extraction method (AOAC method 920.39), while crude fiber was quantified through an AOCS-approved procedure (Ba6a-05). Ash (AOAC method 942.05) and moisture (AOAC method 930.15) contents were measured by gravimetric methods, and available carbohydrates were calculated by difference. Reducing sugars were quantified using a colorimetric method with 3,5-dinitrosalicylic acid [58]. All measurements were conducted in triplicate, and the results are presented as percentages.

Regarding physicochemical parameters, total soluble solids (°Brix) were quantified using a portable refractometer (PAL87S, Atago, Co., Ltd., Tokyo, Japan) by placing a drop of fresh fruit juice directly onto the prism of the instrument. For pH determination, 20 g of fresh fruit was homogenized using a Nutribullet^®^ blender (Nutribullet LLC, Los Angeles, CA, USA). The homogenate was filtered through cotton gauze, and the pH was measured by immersing the electrode of a previously calibrated pH meter (HI 207, HANNA, Bedford, UK) directly into the filtrate. Titratable acidity was determined by the pH-titration method (AOAC method 942.15) [57]. Briefly, 5 mL of the filtrate obtained for pH determination was mixed with 25 mL of distilled water and titrated with 0.1 M NaOH to a pH of 8.3 using a calibrated pH meter; the results were calculated as a percentage of citric acid. Titratable acidity was calculated using the following equation (Equation (1)).

(1) $$Titratable\;acidity\left(\%\;citric\;acid\right)=\left(\frac{V\times N\times mEq}{Sample\;volume}\right)\times 100$$

where “V” is the volume of NaOH used (mL), “N” is the normality of NaOH, and “mEq” is the milliequivalent of citric acid (0.064 g/mEq).

Color was measured using a portable colorimeter (FRU, WR10QC, Shenzhen, China) on the CIELab scale. The sample was placed in a glass sample cup, and the colorimeter was positioned directly on the sample surface for measurement according to the manufacturer’s instructions. The activity water (a_w_) was measured using portable equipment (VTSYIQI, VTS160A, Hefei, China). The sample was placed in the manufacturer’s sample cup, and measurement was performed according to the manufacturer’s specifications. All measurements were conducted in triplicate.

For functional properties, the water solubility index (WSI, %) and water absorption index (WAI, g g^−1^) were measured employing the methodology described by Anderson et al. [59]. Briefly, the sample (2.5 g) was dispersed in distilled water (30 mL), vortex-mixed for 1 min, and incubated at 30 °C under continuous agitation (80 rpm) for 30 min. The suspension was subsequently centrifuged (Hermle Z32HK, Wehingen, Germany) at 6000 rpm for 10 min to separate the soluble and insoluble fractions, The supernatant was recovered and evaporated (100 °C for 24 h) to constant weight, and the recovered soluble solids were used to calculate WSI (Equation (2)), whereas WAI was calculated from the weight of the hydrated sediment after centrifugation (Equation (3)). All measurements were conducted in triplicate.

(2) $$Water\;solubility\;index\left(\%\right)=\left(\frac{Weight\;of\;pellet\;after\;centrifugation}{Weight\;of\;sample}\right)\times 100$$

(3) $$Water\;absorption\;index\left(g/g\right)=\left(\frac{Weight\;of\;the\;pellet\;after\;centrifugation}{Weight\;of\;the\;sample-Weight\;of\;the\;pellet\;after\;centrifugation}\right)\times 100$$

The oil absorption capacity was determined as recommended by Nasrin et al. [60], using corn oil (g g^−1^). Briefly, 2.5 g of dry sample was mixed with 30 mL of corn oil, vortex-mixed for 1 min, and centrifuged at 10,000 rpm for 60 min. The supernatant was carefully decanted, and the weight of the resulting sediment was recorded. The results were calculated from the weight difference and expressed as grams of oil absorbed per gram of dry sample. The swelling power (%) was determined following the gravimetric method described by Uarrota et al. [61]. A dried fruit sample (100 mg) was mixed with 10 mL of distilled water and incubated in a water bath at 60 °C for 30 min. After cooling to room temperature, the suspension was centrifuged at 3000 rpm for 30 min. The weight of the hydrated sediment was used to calculate the swelling power after correcting for the sample’s initial moisture content, using the following Equation (4). All measurements were conducted in triplicate.

(4) $$Swelling\;power\left(\%\right)=\left(\frac{Weight\;of\;the\;sediment}{Weight\;of\;the\;sample\;with\;moisture\;corrected}\right)\times 100$$

Emulsifying capacity (%) was determined using the method described by Yasumatsu et al. [62], with canola oil. Briefly, dried *F. tomentosa* fruit (2 g) were combined with distilled water (20 mL) and canola oil (20 mL), and the mixture was homogenized at 70 rpm for 20 min. After centrifugation at 10,000 rpm for 10 min, the height of the emulsion layer was measured using a digital vernier caliper (Steren HER-411, Shanghái, China), and the results were calculated using Equation (5). Foaming capacity (%) was determined following the methodology described by Piornos et al. [63]. A 1% (*w*/*v*) suspension of *F. tomentosa* dried fruit in distilled water was prepared and vortex-mixed for 1 min. The suspension was subjected to high-speed homogenization (LANKAI FSH-2A, Wuhan Baykul Electronic Commerce Co., Ltd., Wuhan, China) at 12,500 rpm for 60 s at room temperature. The height of the foam layer formed after homogenization was measured with a digital Vernier caliper, and the results were calculated using Equation (6). All measurements were conducted in triplicate.

(5) $$Emulsifying\;capacity\left(\%\right)=\left(\frac{Height\;of\;emulsified\;layer}{Height\;of\;whole\;layer\;in\;the\;conical\;tube}\right)\times 100$$

(6) $$Foaming\;capacity\left(\%\right)=\left(\frac{Height\;of\;the\;suspension\;after\;homogenization\left(mm\right)-Height\;of\;the\;suspension\;before\;homogenization(mm)}{Height\;of\;the\;suspention\;before\;homogenization(mm)}\right)\times 100$$

### 5.3. Soluble Phenols, Flavonoids, Anthocyanins, Condensed Tannins, and Antioxidant Capacity

To quantify the soluble phenols, flavonoids, condensed tannins, and antioxidant capacity, an extraction was performed on fruit powder (0.5 g dried mass) mixed with a 80:20 methanol–acidified water (10 mL) solution (0.8% HCl, 72.8 g/L) [64,65]. The quantification of soluble phenols was based on the method described by Montreau [66], using the Folin–Ciocalteu colorimetric assay at 750 nm and a calibration curve of gallic acid (R^2^: 0.998), and expressed as milligrams of gallic acid equivalents per gram (mg GAE/g). For this, extract (12 µL) was mixed with 2N Folin–Ciocalteau solution (12 µL), 116 of 7.5% *w*/*v* Na2CO3, and distilled water (164 µL) in a 96-well microplate and incubated under agitation in an orbi-shaker (Benchmark, Orbi-Shaker 4-Plate BT1502, Sayreville, NJ, USA) at 200 rpm for 15 min in the dark.

The flavonoid content was determined by the aluminum chloride colorimetric assay at 510 nm, with catechin as the standard (R^2^: 0.999), and expressed as milligrams of catechin equivalents per gram (mg CE/g) [67]. Briefly, *F. tomentosa* fruit extract (100 µL) was combined with 5% (*w*/*v*) sodium nitrate solution (430 µL) and allowed to stand for 5 min. Subsequently, 10% (*w*/*v*) aluminum chloride solution (300 µL) was added, and the reaction mixture was maintained for an additional minute. Then, 1 M sodium hydroxide solution (440 µL) was incorporated, and the mixture was vortexed. An aliquot (200 µL) of the resulting reaction mixture was transferred to a 96-well microplate, and the absorbance was recorded.

The condensed tannins were determined using the vanillin–HCl method at 500 nm with a catechin calibration curve (R^2^: 0.998), and the results were expressed as milligrams catechin equivalents per gram (mg CE/g) [68]. In a test tube, *F. tomentosa* fruit extract (133 µL) was combined with 1 mL of 4% (*w*/*v*) vanillin in methanol and 500 µL of concentrated hydrochloric acid. After vortex mixing for 30 s, the reaction mixture was kept in the dark at 30 °C for 15 min. Subsequently, 200 µL of the resulting reaction mixture was transferred to a 96-well microplate, and the absorbance was recorded. On the other hand, to determine anthocyanin content, a new extraction was performed by mixing fruit powder (500 mg) with 10 mL of an acidified ethanol solution (85:15, *v*/*v*, ethanol and 1 N HCl, adjusted to pH 1). The mixture was magnetically stirred for 30 min and centrifuged at 3200 rpm. The resulting supernatant was transferred to a 10 mL volumetric flask and brought to volume with acidified ethanol. The absorbance was measured at 535 nm, and a calibration curve for cyanidin-3-O-glucoside was constructed (R^2^: 0.999), with results expressed as milligrams of cyanidin-3-O-glucoside equivalents per gram (mg C3OE/g) [69].

The antioxidant capacity was determined in the methanolic-water extracts. ABTS (2,2-azinobis-3-ethylbenzothiazoline-6-sulfonic acid) radical scavenging capacity was determined by combining *F. tomentosa* fruit extract (35 µL) with 7 mM ABTS working solution (265 µL) in a 96-well microplate. The reaction mixture was incubated in the dark with agitation at 200 rpm for 10 min, after which the absorbance was recorded at 734 nm [70]. DPPH (2,2-diphenyl-1-picrylhydrazyl) radical scavenging capacity was determined by mixing *F. tomentosa* fruit extract (40 µL) with 190 µM DPPH working solution (260 µL) in a 96-well microplate. The reaction mixture was incubated in the dark with agitation at 200 rpm for 30 min, after which the absorbance was recorded at 517 nm [71]. Ferric-reducing power (FRAP) was evaluated by combining *F. tomentosa* fruit extract (36 µL) with 264 µL FRAP working solution [0.3 M acetate buffer (pH 3.6), 20 mM FeCl_3_, and 10 mM TPTZ at a 10:1:1 (*v*/*v*/*v*) ratio], and distilled water (9 µL) in a 96-well microplate, and the reaction mixture was maintained in the dark with agitation at 200 rpm for 30 min, after which the absorbance was recorded at 595 nm [72]. In all antioxidant capacity assays, Trolox (6-hydroxy-2,5,7,8-tetramethylchroman-2-carboxylic acid) was used to construct standard calibration curves (R^2^: 0.999, 0.992, and 0.997, respectively), and results were expressed as Trolox-equivalent millimoles per gram (mmol TE/g).

All quantifications of this section were performed using a microplate reader (ACCURIS, SmartReader MR-9600, Accuris Instruments, Nanjing, China). All measurements were conducted in triplicate.

#### Phenolic Profile by HPLC-DAD

Phenolic compounds were identified and quantified by high-performance liquid chromatography using an Agilent 1260 Infinity HPLC system (Santa Clara, CA, USA) equipped with a diode-array detector (DAD) and a Poroshell 120 EC-C18 reversed-phase column (4.6 × 100 mm, 2.7 μm), following the methodology previously reported by [73] without modifications. The phenolic extract (Section 5.3) was concentrated under reduced pressure at 35 °C using a rotatory evaporator (Yamato RE300, Tokyo, Japan), reconstituted in 1 mL of methanol, and filtered through a 0.22 µm membrane before injection (10 μL). The mobile phase consisted of solvent A (0.1% trifluoroacetic acid in HPLC-grade water, *v*/*v*) and solvent B (acetonitrile), delivered at a flow rate of 0.5 mL/min using the following gradient: 0–10 min, 100% A; 10–15 min, 80% A and 20% B; 15–20 min, 75% A and 25% B; 20–35 min, 65% A and 35% B; 35–55 min, 25% A and 75% B; 55–57 min, 100% B; 57–62 min, 35% A and 65% B; 62–65 min, 65% A and 35% B, and 65–70 min, 100% A. The DAD monitored wavelengths between 270 and 320 nm, with phenolic acids detected at 270 nm, flavonoids between 270 and 280 nm, and hydroxybenzoic and hydroxycinnamic acids within the 280–320 nm range. Identification and quantification were performed using phenolic standards (Sigma-Aldrich, St. Louis, MO, USA), and the results were expressed as μg/100 g dried weight. All measurements were conducted in triplicate. Method validation data, including a representative HPLC-DAD chromatogram, calibration curve equations, coefficients of determination (R^2^), limits of detection (LOD), limits of quantification (LOQ), and information for each phenolic standard (CAS number and calibration range), are provided in the Supplementary Material (S2, Figure S1 and Table S1).

### 5.4. Toxicity Assay on Artemia salina

The *Artemia salina* assay was conducted in accordance with the methodology outlined by Castorena-Sánchez et al. [15]. *A. salina* cysts were incubated for 24 h at 28 °C in artificial seawater (30 g/L NaCl), under continuous illumination and aeration. Subsequently, ten *A. salina* nauplii (deposited in 5 mL of artificial seawater) were exposed to varying concentrations of *F. tomentosa* fruit extract (62.5, 125, 250, 500, 750, and 1000 µg/mL) in a six-well culture plate. The organisms were maintained in darkness, and after 24 h and 48 h exposure to *F. tomentosa* extract the survival rate was assessed using Equation (7). Sodium hypochlorite was employed as a positive control, while artificial seawater served as a negative control. To enhance visibility during counting, the larvae were observed under a light source to provide contrast. Results were obtained from three independent experiments and measured in triplicate.

(7) $$Survival\left(\%\right)=\left(1-\frac{Dead\;naupli\;in\;the\;bioassay}{Total\;naupli\;at\;the\;begining}\right)\times 100$$

### 5.5. Computational Prediction of Physicochemical, ADME, and Toxicological Properties

To the understand different characteristics of the six most relevant compounds identified in *F. tomentosa* by HPLC-DAD, the SMILES (Simplified Molecular Input Line Entry Specification) notation was retrieved from the PubChem database (https://pubchem.ncbi.nlm.nih.gov/compound/1548943; accessed on 5 March 2026) [74]. The physicochemical properties of the compounds were evaluated using SwissADME (https://www.swissadme.ch/; accessed on 3 March 2026) [39]. Furthermore, the ADME (absorption, distribution, metabolism, and excretion) and toxic traits of the compounds in the extract were examined using the ADMET-AI (https://admet.ai.greenstonebio.com/; accessed on 3 March 2026) [75]. Subsequently, to predict the LD_50_ and toxicity class of the compounds, the ProTox-3.0. (https://tox.charite.de/protox3/index.php?site=compound_input; accessed on 3 March 2026) server was employed [40].

Additionally, molecular docking was performed between the previously mentioned compounds and cytochrome P450 isoforms to explore the structural features of compounds present in *F. tomentosa* that might affect the activation of xenobiotic metabolic pathways. The chemical structures of each compound, including the controls, were retrieved in SDF format from the PubChem database (https://pubchem.ncbi.nlm.nih.gov/; accessed on 24 April 2026) [74]. The three-dimensional crystal structures of human CYP3A4 (PDB ID: 4D6Z), CYP2D6 (PDB ID: 4WNV), and CYP2E1 (PDB ID: 3E6I) were obtained from the Protein Data Bank (https://www.rcsb.org/; accessed on 24 April 2026). Prior to docking, the protein structure was prepared using PyMOL (v.3.1). Molecular docking simulations were carried out using CBDOCK2 (http://183.56.231.194:8001/cb-dock2/index.php; accessed on 24 April 2026) [76]. Before docking, the coordinates of the cavities were obtained using the “Search Cavities” function in the CBDOCK2 platform. The coordinates (x, y, and z) of the cavities used for CYP3A4, CYP2D6, and CYP2E1 were 14, 35, and −9; 5, −23, and 66; and 30, 36, and 16, respectively. The active site of the proteins explored was defined by the position of the co-crystallized ligand (control) in the PDB structure. Subsequently, the interactions between the ligands and proteins were visualized in BIOVIA Discovery Studio.

## 6. Conclusions

This study provides the first comprehensive characterization of *Forestiera tomentosa* fruit, demonstrating its potential as an underutilized native fruit with nutritional and functional value. Moreover, the results revealed that *F. tomentosa* fruit is a rich source of bioactive phenolic compounds, with 20 phenolic constituents identified that contribute to its antioxidant capacity. Additionally, the absence of acute toxicity in the *Artemia salina* assay, together with favorable in silico ADMET predictions, supports its potential safety for further investigations and applications in food-related products.

Future studies should focus on the complete nutritional characterization of this fruit, including its mineral and vitamin profiles and the assessment of antinutritional factors. Furthermore, the isolation and characterization of additional bioactive compounds, together with in vivo studies, are needed to validate their biological activities and to assess potential interactions between bioactive compounds from *F. tomentosa* and therapeutic agents.

## Figures and Tables

**Table 1:** Proximal composition of *Forestiera tomentosa* fruit.

Parameter	Content (%)
* Proteins	7.39
* Lipids	4.30
^†^ Available carbohydrates	71.94
* Crude fiber	7.90
** Reducing sugars	21.40 ± 0.05
* Ash	4.33
* Moisture in dried fruits	12.04
^▲^ Moisture in fresh fruits	67.85 ± 0.12

* Values correspond to the mean values reported by an accredited external analytical laboratory and presented on a dry-matter basis (Supplementary Material, S1). ** Results are mean ± standard deviation of triplicate determinations. ^†^ Results were calculated using average dry-matter-basis data; ▲ Results are presented on a wet-matter basis.

**Table 2:** Physicochemical parameters and functional properties of *Forestiera tomentosa* fruit.

Parameter	Content
Physicochemical parameters	
* pH	5.66 ± 0.02
* Titratable acidity (% citric acid)	0.32 ± 0.07
* Total soluble solids (TSS, °Brix)	2.33 ± 0.01
TSS/TA ratio	7.28
* Luminosity	26.26 ± 1.61
* *a* (coordinate)	1.01 ± 0.35
* *b* (coordinate)	−15.30 ± 0.53
Water activity	0.42 ± 0.01
Functional properties	
Water solubility index (%)	56.10 ± 0.19
Water absorption index (g g^−1^)	1.80 ± 0.24
Swelling power (%)	2.48 ± 0.11
Oil absorption index (g g^−1^)	4.54 ± 0.07
Emulsifying capacity (%)	2.61 ± 0.19
Foaming capacity (%)	19.71 ± 0.89

Results are the mean ± standard deviation of triplicate determinations. Results are presented on a dry-matter basis. * Results are presented on a fresh basis.

**Table 3:** Phenolic compounds content and antioxidant capacity of *Forestiera tomentosa* fruit.

Parameter	Content
Phenolic compounds	
Total soluble phenols (mg GAE/g)	280.42 ± 7.84
Total flavonoids (mg CE/g)	98.89 ± 9.39
Total anthocyanins (mg C3G/g)	54.53 ± 3.07
Condensed tannins (mg CE/g)	94.95 ± 2.05
Antioxidant capacity	
DPPH (mmol TE/g)	14.59 ± 0.26
ABTS (mmol TE/g)	12.04 ± 0.13
FRAP (mmol TE/g)	1.92 ± 0.29

Results are mean ± standard deviation of triplicate determinations. GAE: Gallic acid equivalent; CE: catechin equivalent; C3G: Cyanidin-3-glucoside. All results are presented on a dry-matter basis.

**Table 4:** HPLC-DAD phenolic profile of *Forestiera tomentosa* fruit.

Compound	Content (µg/100 g DW)
Gallic acid	2411.98 ± 340.76
Protocatechuic acid	730.55 ± 6.70
Gallocatechin	23,693.03 ± 196.43
Neochlorogenic acid	3496.48 ± 3.14
3,4-Dihydroxyphenylacetic acid	2696.84 ± 10.92
4-Hydroxybenzoic acid	1876.46 ± 22.27
Chlorogenic acid	1330.67 ± 4.03
4-Hydroxyphenylacetic acid	9229.56 ± 70.98
Vanillic acid	3691.48 ± 40.14
Syringic acid	55,791.95 ± 32.82
Caffeic acid	882.44 ± 3.63
Catechin	29,630.66 ± 122.63
Epicatechin	25,387.03 ± 69.41
3-(4-hydroxyphenyl) propionic acid	172,711.40 ± 63.68
Rutin	2621.29 ± 55.37
*p*-Coumaric acid	3670.09 ± 13.89
Trans-ferulic acid	824.35 ± 5.27
Ellagic acid	2507.57 ± 1.86
Salicylic acid	1158.56 ± 22.31
Naringenin	1730.70 ± 6.52

Results are mean ± standard deviation of triplicate determinations. DW: Dry weight.

**Table 5:** Bioassay in *Artemia salina* larvae after 24 and 48 h of exposure to *Forestiera tomentosa* aqueous extract.

Concentration (µg/mL)	Survival Rate (%)	
	24 h	48 h
1000	100	100
500	100	100
250	100	100
125	100	100
62.5	100	100
1000	100	100
Synthetic seawater (Negative control)	100	100
Sodium hypochlorite solution at 10% (positive control)	0	0

Results are the mean of three independent experiments (*n* = 3). Toxicity was evaluated using a viability scale: 100–91% non-toxic, 90–51% slightly toxic, 50–11% toxic, and 10–0% highly toxic [15].

**Table 6:** In silico analysis of physicochemical and ADME properties of six major phytochemicals identified in *Forestiera tomentosa* fruit.

Compound	MW	nHBA	nHBD	LP	nLR	OB *	HIA **	BBB ***
Syringic acid	198.17	5	2	1.54	0	0.73	0.99	0.43
Catechin	290.27	6	5	1.33	0	0.40	0.99	0.14
Epicatechin	290.27	6	5	1.47	0	0.39	0.99	0.13
Gallocatechin	306.27	7	6	1.47	1	0.35	0.98	0.09
3-(4-Hydroxyphenyl) propionic acid	166.17	3	2	1.19	0	0.79	0.98	0.45
4-Hydroxyphenylacetic acid	152.15	3	2	0.88	0	0.79	0.95	0.45

MW: Molecular weight (g/mol); nHBA: hydrogen-bond acceptors; nHBD: hydrogen-bond donors; LP: lipophilicity; nLR: number of violations of Lipinski’s rule of five; HIA: human intestinal absorption; OB: oral bioavailability; BBB: blood–brain barrier. * A value closer to 1 indicates the capacity of the compound to be absorbed from the human gastrointestinal system into the bloodstream of the human body. ** A value closer to 1 indicates better compound performance in terms of absorption and availability at the site of action. *** A value closer to 1 indicates a higher probability that the compound can cross the BBB.

**Table 7:** In silico analysis of toxicological properties of *F. tomentosa* fruit compounds.

Compound	Clinical Toxicity	hERG Blocking	Drug-Induced Liver Injury	Mutagenicity *	Carcinogenicity **	Predicted LD_50_ (mg/kg)	Toxicity Class
Syringic acid	0.01	0.01	0.69	0.05	0.05	1700	4
Catechin	0.06	0.25	0.26	0.34	0.05	10,000	6
Epicatechin	0.06	0.23	0.25	0.33	0.05	10,000	6
Gallocatechin	0.08	0.30	0.34	0.33	0.04	10,000	6
3-(4-Hydroxyphenyl) propionic acid	0.05	0.01	0.32	0.05	0.22	2000	4
4-Hydroxyphenylacetic acid	0.06	3.71 × 10^−3^	0.30	0.05	0.19	1550	4

* Value closer to 1 indicates a higher probability that the compound produces the effect. ** Toxicity is classified as 1–6; class 1 is the most toxic and lethal, while class 6 is non-toxic.

**Table 8:** Detailed molecular docking information for *Forestiera tomentosa* compounds with cytochrome P450 isoforms.

Compound	PubChem CID	CYP3A4 (kcal/mol)	CYP2D6 (kcal/mol)	CYP2E1 (kcal/mol)
Syringic acid	10742	−6.3	−6.0	−6.4
Catechin	9064	−8.3	−7.7	−9.2
Epicatechin	72276	−8.8	−7.8	−7.9
Gallocatechin	65084	−8.4	−7.5	−9.0
3-(4-Hydroxyphenyl) propionic acid	10394	−6.4	−6.8	−6.9
4-Hydroxyphenylacetic acid	127	−6.6	−6.3	−6.7
Control for CYP3A4 (Tert-Butyl {6-Oxo-6-[(Pyridin-3-Ylmethyl)amino]hexyl}carbamate)	91885508	−7.5	---	---
Control for CYP2D6 (quinine)	3034034	---	−9.0	---
Control for CYP2E1 (Indazole)	9221	---	---	−6.0

## Data Availability

Data are available upon reasonable request from the corresponding author.

## Supplementary Materials

The following supporting information can be downloaded at https://www.mdpi.com/article/10.3390/molecules31142542/s1.

## Abbreviations

ADMET	Absorption distribution, metabolism, excretion and toxicity
AOAC	Association of Official Analytical Communities
a_w_	Activity water
GAE	Galic acid equivalents
CE	Catechin equivalents
C3OE	Cyanidin-3-O-glucoside
ABTS	2,2-azinobis-3-ethylbenzothiazoline-6-sulfonic acid
DPPH	2,2-diphenyl-1-picrylhydrazyl
FRAP	Ferric-reducing power
DW	Dry weight
TROLOX	6-hydroxy-2,5,7,8-tetramethylchroman-2-carboxylic acid
TE	Trolox equivalent
HPLC-DAD	High-performance liquid chromatography with diode array detection
SMILES	Simplified molecular input line entry specification
LD	Lethal dose
TA	Titratable acidity
TSS	Total soluble solids
MW	Molecular weight
nHBAs	Hydrogen-bond acceptors
nHBDs	Hydrogen-bond donors
LP	Lipophilicity
nLR	The number of violations of Lipinski’s rule of five
HIA	Human intestinal absorption
OB	Oral bioavailability
BBB	Blood–brain barrier
WlogP	Water partition coefficient
TPSA	Topological polar surface area
hERG	Human ether-à-go-go-related gene
