# Supplementary material for: Characterization of Forestiera tomentosa Fruit: Proximate Composition, Physicochemical Parameters, Phenolic Content, Antioxidant Capacity, and Toxicological Assessment
Source: Molecules. 2026 Jul 22;31(14):2542. doi: 10.3390/molecules31142542 (PMC13415156; doi:10.3390/molecules31142542)
Supplement: Supplementary file 1 [file molecules-31-02542-s001.zip › molecules-4412806-supplementary.pdf]

Previlab

Premezclas y Vitaminas Tepa S. A. de C. V.
Laboratorio de Control de Calidad

Previtep

INFORME DE ENSAYOS

‡29466-1(Š

No. de muestra: 29466-1

13 DE AGOSTO DEL 2025

CLIENTE NUEVO
AV. JALISCO 840 LAS AGUILILLAS TEPATITLAN DE MORELOS JALISCO TEPATITLAN DE MORELOS , 47698 Tel.
Atención: SALVADOR HERNANDEZ ESTRADA

DATOS DE MUESTREO E IDENTIFICACIÓN

Fecha/Hora/Lugar de Muestreo:	06/08/25
Fecha/Hora Recepción Muestras:	06/08/25 15:38
Identificación de la Muestra:	GRANJENO
Matriz de la muestra:	No declarado
Muestreado por/Muestreador/Tipo de Muestreo:	CLIENTE NUEVO / /
Fecha de Preparación:	
Fecha de caducidad:	

Fisicoquímicos:

PARÁMETRO	RESULTADO	UNIDADES	MÉTODO	ANALIZADO POR		AA
Cenizas	4.33	%	AOAC 942.05	11/08/25	JRJ	CC077
Fibra Cruda	7.90	%	AOCS Approved Procedure Ba6a-05	11/08/25	EVR	C.C.077
Grasa	4.30	%	AOAC 920.39	08/08/25	MAV	C.C.077
Humedad	12.04	%	AOAC 930.15	13/08/25	JRJ	C.C.077
Proteína Cruda N x 6.25	7.39	%	AOAC 968.06	08/08/25	EVR	C.C.077

SIMBOLOGÍA:

----- No. Aplica
< LD Menor al Límite Detectable
NMP Número mas probable
N.E. No Efectuado
L.M.P. Límite Máximo Permisible
AA Acreditaciónes y Aprobaciones

NOMENCLATURA:

Estos resultados solo aplican a la muestra recibida.
Técnicas de análisis basadas en las normas de la AOAC y NMX.

Atentamente
MVZ. Juan E. Carlos Molás Mojica
Jefe de Laboratorio de Control de Calidad

Av. Jalisco 840 I Col. Las Aguilillas I Tepatitlán, Jalisco, México
C.P. 47698 I Tel/Fax (378) 781-0730 I www.previtep.com.mx

Supplementary Materials S2

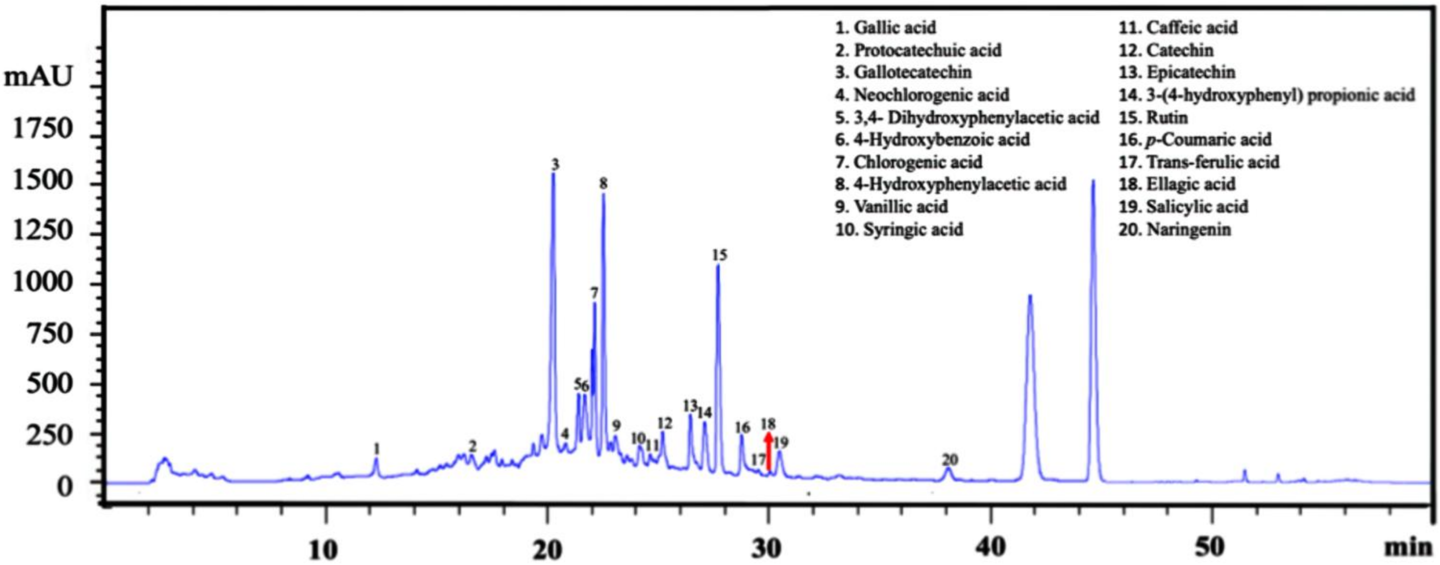

Figure S1. Example of an HPLC-DAD chromatogram at 270 nm of phenolic compounds from *Forestiera tomentosa* fruit.

Table S1. Detection and quantification limits of the standards for phenolic compounds detected.

Phenolic compounds	CAS Number	Purity	Concentration (µg/ml)	R-squared	Formula	Detection limit (LOD, µg/ml)	Quantification limit (LOQ, µg/ml)
Shikimic acid	138-59-0	≥99%	156.25-2500	0.9967	y= 1.3354x-2.52	0.494	1.50
Ellagic acid	476-66-4	≥95%	3.13-50	0.9986	y=591.73x+411.55	0.027	0.08
Gallic acid	5995-86-8	≥99%	6.25-100	0.9979	y=262.9x-402.91	0.071	0.21
Protocatechuic acid	99-50-3	≥99%	3.13-50	0.9997	y=391.51x-112.28	0.057	0.17
Neochlorogenic acid	906-33-2	≥98%	12.5-200	0.9984	y=288.88x-947.31	0.085	0.26
3,4-dihydroxyphenylacetic acid	102-32-9	98%	3.13-50	0.9987	y=117.31x-91.121	0.118	0.36
4-hydroxybenzoic acid	99-96-7	≥99%	3.13-50	0.9989	y=376.93x-287.21	0.086	0.26
Chlorogenic acid	327-97-9	≥95%	6.25-100	0.9992	y=389.28x-533.98	0.056	0.17
Vanillic acid	121-34-6	≥97%	6.25-100	0.9997	y=286.35x-300.01	0.062	0.19
Syringic acid	530-57-4	≥95%	6.25-100	0.9993	y=395.91x-320.34	0.076	0.23
Caffeic acid	331-39-5	≥98%	3.13-50	0.9995	y=637.32x-421.92	0.036	0.11
Transcinnamic acid	140-10-3	≥99%	3.13-50	0.9983	y=1070.9x-1271.9	0.024	0.07
Epicatechin	490-46-0	≥98%	6.25-100	0.9997	y=133.9x-130.53	0.204	0.62
4-hydroxybenzaldehyde acid	123-08-0	98%	3.13-50	0.9966	y=1083.8x+415.7	0.017	0.05
Homovanillic acid	306-08-1	>99%	3.13-50	0.9997	y=90.436x-42.411	0.340	1.03
3-(4-hydroxyphenyl) propionic acid	501-97-3	98%	6.25-100	0.9991	y=47.712x-21.511	0.320	0.97
Rutin	207671-50-9	≥94%	3.13-50	0.9998	y=137.5x-51.767	0.170	0.51
p-coumaric acid	501-98-4	≥98%	6.25-100	0.9695	Y=496.93x+1844.1	0.043	0.13
Trans-ferulic acid	537-98-4	99%	3.13-50	0.9984	y= 343.49x-328.28	0.094	0.28
Galocatechin	3371-27-5	≥98%	6.25-300	0.998	y=15.557x-26.384	0.902	2.73
Epigallocatechin	970-74-1	≥95%	6.25-100	0.9995	y=23.341x-31.289	0.553	1.68
Catechin	18829-70-4	≥97%	3.13-50	0.9992	y=84.567x-63.346	0.222	0.67
Mirycetin	529-44-2	≥96.0%	3.13-50	0.9995	y=298.06x-154.5	0.075	0.23
Naringenin	67604-48-2	98%	3.13-50	0.9995	y=587.5x-304.69	0.054	0.16
Hydroquinone	123-31-9	≥99%	3.13-50	0.9981	y=174.19x-57.191	0.145	0.44
2,5-dihydroxybenzoic acid	490-79-9	98%	3.13-50	0.9976	y=12.891x-7.9086	0.842	2.55
Salicylic Acid	69-72-7	≥99.0%	3.13-50	0.9996	y=250.15x-121.13	0.068	0.21

trans-2-Hydroxycinnamic acid	614-60-8	97%	3.13-50	0.9916	y=947.49x-1115.7	0.031	0.10
Quercetin	117-39-5	≥95%	3.13-50	0.9992	y=352.38x-281.21	0.064	0.19
